# Impaired Attention Functioning in Children and Adolescents with Obesity: Preliminary Results Based on the Computerized Continuous Performance Test

**DOI:** 10.3390/jcm14248656

**Published:** 2025-12-06

**Authors:** Katarzyna Anna Majewska, Maia Stanisławska-Kubiak, Paulina Wais, Joanna Budzulak, Ewa Mojs, Andrzej Kędzia

**Affiliations:** 1 Department of Pediatric Diabetes, Auxology and Obesity, Poznan University of Medical Sciences, 61-701 Poznan, Poland; paula.wyrwas@gmail.com (P.W.); j.budzulak@gmail.com (J.B.); akedzia@ump.edu.pl (A.K.); 2 Department of Clinical Psychology, Poznan University of Medical Sciences, 61-701 Poznan, Poland; maiakubiak@gmail.com (M.S.-K.); ewamojs@ump.edu.pl (E.M.)

**Keywords:** attention, obesity, children, cognitive functions, continuous performance test

## Abstract

**Background/Objectives:** Attention is a fundamental cognitive parameter, essential for developmental processes. It enables the selective processing of environmental stimuli and guides behavioral responses. Obesity, apart from its broad influence on human somatic health, is also associated with mental and cognitive functioning. In childhood obesity, detailed attention assessment could help elucidate the relationship between the condition and cognitive development, and perhaps also help predict specific difficulties during treatment. The aim of the study was to investigate attention functioning in children and adolescents with obesity using the computerized continuous performance test (CPT). **Methods:** The study involved 71 children, including 23 with obesity and 48 healthy children with normal body weight. The MOXO CPT was used to assess attention parameters in all participants. The test covered four parameters: sustained attention, timing, impulsivity, and hyperactivity. **Results:** Children with obesity obtained significantly lower CPT results in terms of timing (*p* = 0.024), hyperactivity (*p* = 0.001), and impulsivity (*p* < 0.001), while the difference in sustained attention did not reach statistical significance (*p* = 0.074). **Conclusions:** Attention efficiency appears to be reduced in children with obesity compared with their healthy peers. Assessment of attention parameters in this group of patients could be valuable in the context of planning and implementing therapeutic interventions. Children with coexisting obesity and impaired attention functioning would probably require more assistance in following daily behavioral and nutritional recommendations.

## 1. Introduction

Advances in research on cognitive processes, including neuroimaging techniques and experimental psychology tests, have proven to be useful in understanding cognitive functioning. This particularly applies to neurodevelopmental disorders (such as ADHD and autism) and executive function disorders. Thanks to these advances, we can expand our understanding of cognitive components and their role in performing specific tasks [1,2,3,4]. Of particular interest are the executive functions, representing the highest level of cognitive control. They are crucial for goal setting, planning, task execution, and behavioral adaptation. These functions facilitate the learning of new skills, error correction, the initiation of new action sequences, and conscious control over behavior, enabling flexible responses to changing environments [5,6,7]. The review of concepts and empirical studies allows us to distinguish the core processes in the structure of executive functions: cognitive flexibility (shifting), which allows the rapid adaptation to changing stimuli; inhibitory control (inhibition), which enables the suppression of automatic responses in favor of alternative actions; and working memory, which is essential for storing and manipulating information needed for ongoing tasks [7,8].

Attention is a fundamental cognitive parameter, essential for developmental processes. It enables the selective processing of environmental stimuli and guides behavioral responses. Attention is inherently active, not only filtering relevant information but also exerting control over cognitive processes. It facilitates focus on specific objects, sustains engagement over time, and differentiates significant stimuli from those that are irrelevant. It is essential for the proper functioning of most cognitive processes. A key characteristic of attention is its limited capacity—concentrating on one stimulus may inhibit processing of others, which may still be registered at a preattentive level, automatically and without conscious awareness. Additionally, attention requires cognitive effort, and prolonged focus on a single stimulus can lead to fatigue. Thus, its efficiency is measured not only by the ability to distinguish relevant from irrelevant stimuli but also to sustain focused attention over time. Attention is not a unitary construct; its assessment requires tasks evaluating selectivity, sustained alertness, and attentional shifting [7,8,9].

Obesity, by definition, is a condition of excessive fat accumulation that causes a risk of severe and chronic health consequences. It is considered a significant medical problem of the developmental period, as approximately 160 million children and adolescents aged 5 to 19 live with obesity, along with another 35 million of those aged below 5. It has been assessed that the obesity rate in adolescents has quadrupled since 1990 [10]. In addition to global trends, recent large population-based studies conducted in Poland have confirmed a marked and systematic rise in the prevalence of overweight and obesity, also within the Polish pediatric population [11,12]. This is particularly worrying in the context of future serious health complications, especially in the field of metabolic disorders, such as type 2 diabetes, dyslipidemia, atherosclerosis, hypertension, or metabolic dysfunction-associated fatty liver disease, but also in terms of psychological difficulties, additionally disturbing the developmental period. Adipose tissue is not only an energy storage site, but also an active endocrine organ that secretes numerous biologically active factors, affecting distant tissues. Adipokines, as they are called, are involved in regulatory processes related to body metabolism, inflammation, insulin sensitivity, cardiovascular health, appetite control, and neural integrity. Scientific evidence indicates that an excess of adipose tissue content alters the profile of adipokine secretion, and thus, related processes may be disrupted [10,13,14,15]. It appears that it may also apply to cognitive functions. An increased body fat mass appears to predict lower cognitive abilities in later adult life, including executive functioning, memory, and learning [16], as well as an increased risk of dementia [10]. A number of studies also present evidence of selective neuropsychological dysfunctions in children with obesity [17].

A broad understanding of the causes and consequences of obesity is essential to effectively fight this worldwide epidemic. From the perspective of its potential links to obesity, attention plays a crucial role in developing stress-coping strategies, acquiring and applying knowledge, assessing problematic situations, and anticipating consequences. Therefore, children with co-occurring obesity and attention disorders may experience significant difficulties in the treatment process, especially regarding long-term adherence to behavioral and nutritional recommendations. In childhood obesity, a detailed assessment of attention could help elucidate the relationship between the condition and cognitive development. Some studies indicate that children and adolescents with obesity tend to perform slightly worse than their peers on tasks requiring sustained attention, rapid processing, memory, and visual-spatial functioning. Recent meta-analyses suggest that this may be particularly relevant to attention and reaction inhibition [7].

The basis of neuropsychological diagnostics is a standardized psychometric test and observation of the patient. Methods of measuring executive functions used in developmental studies typically focus on a single component. These are simple tasks, adapted to the abilities of children. They usually refer to the basic form of activity, which is a child at play, and they may also take the form of a computer game. Using tests allows for quantitative recognition of the competences examined using parameters such as reaction time, the number of correct answers, or the proportion of correct and incorrect answers [18]. Moreover, scientific research may also use, in addition to quantitative indicators, a qualitative analysis of the respondents’ behavior, distinguishing categories of behavior and linking them to quantitative indicators [19].

The MOXO continuous performance test (CPT) is a computerized method that measures a child’s neuropsychological functioning in a manner similar to measuring features in biological sciences. It enables an objective assessment and facilitates inter- and intrapersonal comparisons [20].

The aim of the study was to evaluate attention functioning in children and adolescents with obesity using the computerized continuous performance test.

## 2. Materials and Methods

### 2.1. Subjects

In total, 71 Caucasian children participated in this study: 23 with obesity, patients from the Karol Jonscher Memorial Hospital of the Poznan University of Medical Sciences, who formed the study group, and 48 healthy children with normal body weight, who were included in the control group. Detailed group characteristics are presented in Table 1.

### 2.2. Inclusion and Exclusion Criteria

The inclusion criteria for the study group included an isolated obesity diagnosis, with no comorbidities or metabolic complications, and BMI z-score values above 2.0, as well as an age range of 6 to 18 years. For healthy children from the control group, the inclusion criteria consisted of optimal normal weight, with a BMI z-score between −1.0 and 1.0, and an age range of 6 to 18 years. The exclusion criteria for both groups were as follows: age below 6 or above 18 years, any known acute or chronic diseases (other than obesity in the study group), as well as diagnosed or suspected developmental or psychological disorders, including attention deficit hyperactivity disorder (ADHD). Participants were consecutively recruited in the hospital-based settings. All eligible patients attending routine visits during the study period were invited to participate. Participation was voluntary, and no incentives were provided. This approach minimized potential selection bias by avoiding purposive sampling and ensuring that all eligible children had an equal opportunity to participate.

All participants lived in the same geographical region (the Greater Poland province) under similar environmental conditions, in urban areas. All were from families of stable financial condition, attending public educational institutions, and using public health-care services; therefore, their socioeconomic status was considered similar.

### 2.3. Clinical Evaluation

All participants had an exact evaluation of their height and weight. Height measurements were performed using a stadiometer, taken in centimeters (cm) with an accuracy of 0.1 cm. Body weight was measured in kilograms (kg) with an accuracy of 0.1 kg using a medical scale. Based on the collected data, the body mass index (BMI; weight in kilograms/[height in meters]^2^) was calculated for all the children and standardized for age and gender, expressed as a BMI z-score according to Cole’s LMS method [21], following the formula: BMI z-score = [(BMI/M)^L^ − 1]/[L × S] [22,23]. LMS parameters were extracted from references based on the Polish pediatric population [24,25].

In children with obesity, basic laboratory tests were also performed, including serum thyrotropin (TSH), free thyroxine (FT4), cortisol, total cholesterol, alanine aminotransferase (ALT), and aspartate aminotransferase (AST) levels. All patients underwent an oral glucose tolerance test, with evaluation of glycemia before (fasting) and 120 min after administering glucose at a dose of 1.75 g per kg of body weight (maximum 75 g). These test results were used to confirm the absence of basic comorbidities and metabolic complications in the obesity group.

### 2.4. Continuous Performance Test

The MOXO CPT was used to assess attention parameters for all participants.

MOXO (Neuro-Tech Solutions, Hofit, Israel) is a standardized computerized test of attention efficiency. It uses visual and auditory stimuli as distractors, similar to everyday life. The person being tested, under the guidance of the diagnostician, performs tasks that enable the assessment of four indicators of attention: the ability to sustain attention, timing, impulsivity, and hyperactivity. This assessment allows for the creation of a comprehensive attention profile. The ability to sustain attention reflects the patient’s capacity to accurately locate, evaluate, and respond to a stimulus in accordance with the task’s requirements. Timing verifies the ability to react correctly within the time allocated for the task. Impulsivity is the tendency to react at a moment defined as inappropriate. Hyperactivity is the difficulty in effectively regulating motor performance and refraining from unnecessary or undesirable actions [26,27].

MOXO is standardized according to the patient’s age and can be performed in adults, adolescents, and children aged six or older. The average test time is approximately 15–18 min, depending on the patient’s age. During the test, detected reactions are automatically measured and calculated, and further converted into an individual attention profile. Individual test results are reported as standard deviations from the norm, matched for age and gender according to the test references.

All children completed the test in a standardized environment, with no external distractions. Tests were performed during morning hours, after a normal night’s sleep, and one hour after a standard breakfast. Participants did not engage in intense physical activity before testing. After the examination, caregivers of each child received feedback on the test results and possible recommendations for further care, if necessary.

### 2.5. Statistical Analysis

Statistical analysis was performed using IBM SPSS Statistics 29. The value of *p* < 0.05 was considered statistically significant. Quantitative traits have been characterized by applying the mean value and standard deviation (SD). The *t*-test for independent data was used to assess differences between the study group and the control group in terms of gender, while the Chi-square test was used to assess differences in terms of age. The Kolmogorov–Smirnov test was used to evaluate the compatibility of the studied variables with the normal distribution. Due to a lack of normal distribution, the nonparametric Mann–Whitney test was used to determine further differences between the two groups. Additionally, the effect size analysis, using the Glass biserial correlation coefficient, was employed to assess the influence of the independent variables on the dependent variable.

The study had a preliminary character. The available sample size included 23 children with obesity and 48 children in the control group. No a priori sample size calculation was performed; instead, a post hoc power analysis based on the actual group sizes was presented. With α = 0.05 (two-tailed test, two independent groups), the available sample (n_1_ = 23, n_2_ = 48) provides approximately 50% power to detect a moderate effect (Cohen’s d = 0.5) and about 75% power for a large effect (d ≈ 0.7). The power to detect small effects (d ≤ 0.3) is limited.

## 3. Results

Among the 23 children and adolescents with obesity, there were 12 girls (52.2%) and 11 boys (47.8%). The control group covered 20 girls (41.7%) and 28 boys (58.3%). The Chi-square test revealed no significant differences with regard to gender in both groups (χ^2^ = 0.69; *p* = 0.452). The *t*-test for independent data also revealed no differences with regard to age (t = 0.17; *p* = 0.434).

Basic laboratory tests performed in children with obesity were as follows (mean values ± SD): TSH: 2.28 ± 0.96 µIU/mL, FT4: 1.03 ± 0.14 ng/dL; cortisol: 99.9 ± 26.1 ng/mL; total cholesterol: 166.8 ± 19.4 mg/dL; fasting glycemia: 86.8 ± 6.4 mg/dL; glycemia measured 120 min after oral glucose administration: 106.5 ± 29.7 mg/dl; ALT: 24.2 ± 7.1 U/L; AST: 25.0 ± 5.0 U/L.

**Figure 1:**
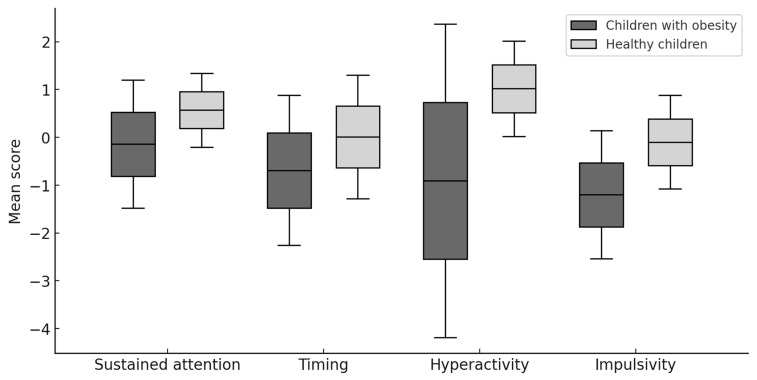
Comparison of MOXO CPT results in children with obesity and healthy children.

The results presented in Table 2 and Figure 1, obtained using the Mann–Whitney U test, showed that children and adolescents with obesity differed significantly from healthy ones in 3 of the analyzed parameters. Children and adolescents with obesity obtained lower CPT performance parameters in terms of timing, hyperactivity, and impulsivity. Additionally, the effect size analysis, which was examined using the Glass biserial correlation coefficient, revealed a moderate influence of the independent variable group on the dependent variables of timing, hyperactivity, and impulsivity.

The results presented in Table 3, obtained using the Mann–Whitney U test, showed that in the group of children with obesity, girls were characterized by greater impulsivity than boys. Additionally, an analysis with the Glass biserial correlation coefficient showed a moderate effect of the independent variable “gender” on the dependent variable “impulsivity”. In the case of the remaining MOXO parameters, no significant differences were detected. Among the children from the control group, no significant differences were found between girls and boys.

In another analysis, girls and boys with obesity were compared according to their appropriate gender with healthy girls and boys (Table 4). The results obtained using the Mann–Whitney U test revealed that all the attention parameters, sustained attention, timing, hyperactivity, and impulsivity, were significantly more disturbed in girls with obesity than in healthy girls. Additionally, the Glass biserial correlation coefficient revealed a significant effect of the independent variable “obesity” on the dependent variable “impulsivity” in girls, as well as a moderate effect of this variable on the dependent variables “sustained attention”, “timing”, and “hyperactivity”. In a similar analysis among boys, the differences between those with obesity and healthy ones did not reach statistical significance.

To supplement the analyses, an additional division was made based on the mean age of the patients, categorizing them into two groups: those below and above 11 years of age. The results presented in Table 5, obtained using the Mann–Whitney U test, showed that in both the group of children with obesity and in the control group, there were no significant differences between younger children up to 11 years of age and older children above 11 years of age in terms of the MOXO parameters examined.

## 4. Discussion

Facing the globally rising incidence of obesity, a multidirectional approach to the problem is necessary, also taking into account the neuropsychological perspective. Obese people often present persistent difficulties in regulating their behavior related to nutritional control and physical activity. Therefore, a wide understanding of the problem should enable more individualized approaches and more effective interventions. A particular group of patients are children, and therapeutic intervention should be started as early as possible to counteract the growing problem of obesity and to obtain optimal effects.

Attention is a multifactorial concept, and it is not entirely clear which of its factors may be most closely associated with obesity. It is defined as the ability to select and concentrate on selected stimuli in an environment where a number of other stimuli are also present; therefore, this cognitive process includes not only the ability to sustain concentration, but also to inhibit the influence of other stimuli [7]. In children with obesity, attention is involved in, among other things, decision-making and the ability to modify diet and physical activity in response to changing daily life circumstances, with the goal of losing weight. The likelihood of making accurate and sound decisions could be related to particular attention disturbances. For instance, difficulties with sustained attention may reflect the patient’s inability to focus on the environment or specific details when required, while a person with timing problems may hesitate when a quick response to environmental changes is necessary. In turn, a person with impulsive tendencies may act without considering the current situation or the possible consequences. Typical features of impulsivity include difficulty with delaying eating, the consumption of high-calorie foods, and engaging in dangerous behaviors without considering the potential consequences [7,28]. Another attention element, hyperactivity, is the difficulty in effectively regulating motor performance and refraining from unnecessary or undesirable actions (such as eating).

Our study confirmed a higher incidence of attention disorders in children with obesity and showed significant abnormalities in three parameters: timing, impulsivity, and hyperactivity. The use of MOXO-CPT enabled us to obtain results that seem consistent with numerous previous studies.

Meta-analyses provide evidence for cross-sectional associations between cognitive functions and obesity. Research indicates that adolescents with obesity tend to perform worse on attention tasks compared to normative data [7,29,30,31]. Children with overweight also exhibit greater difficulties with inhibitory control, often displaying less effective inhibition strategies, particularly when stimuli are food-related [7,32].

Studies on impulsivity in the context of obesity treatment suggest that higher impulsivity levels are associated with greater baseline overweight and poorer weight loss outcomes following intervention. Additionally, impulsivity is linked to attention deficit hyperactivity disorder (ADHD), which may contribute to overeating behaviors and, consequently, to obesity [7,33,34,35].

The review by Liang et al. [7] supports the association between obesity and deficits in executive functioning and attention. Studies show that children with higher body fat, particularly adolescents, tend to perform worse on attention and concentration tasks compared to peers with normal body weight and composition [36,37].

Among executive functions, only certain domains, such as inhibition and delayed gratification, present stronger links to excessive body weight. Others, like decision-making and planning, demonstrate less consistent associations [38], suggesting that different executive functions may influence body mass regulation through distinct mechanisms.

Our study revealed gender-dependent differences in attention parameters within the group of children with obesity—girls presented a higher level of impulsivity than boys, while timing, hyperactivity, and sustained attention did not differ significantly in terms of gender. No corresponding differences were found within the group of healthy children. Interestingly, an in-depth analysis revealed that all attention parameters appeared to be worse in girls with obesity, when compared to healthy girls, especially in terms of impulsivity, while in boys these differences were less pronounced and did not reach statistical significance.

Although many studies of childhood obesity do not consider gender differences, this finding is consistent with some literature suggesting that girls may experience greater psychological distress related to their weight. This distress is often related to social pressure, as girls are subject to more stringent social norms regarding body image compared to boys [39]. Moreover, the methodology used in this study, which involved a computer-based task measuring reaction time, may have introduced an additional layer of complexity for girls. Previous research suggests that such tasks may be more cognitively demanding for girls, generating greater anxiety during performance-based assessments, which in turn could, in theory, affect their scores [40]. Additionally, boys, in general, spend more time playing video games [41]; therefore, we may hypothesize that the computerized test used in our research might have been relatively easier for them to perform than it was for girls.

Understanding the link between neurobiology and obesity may be particularly important in children. It is possible that immature brain processes could contribute to an increased risk of childhood obesity. During normal brain development, areas that perform basic functions, such as motor and sensory systems, mature the earliest; higher-order associative areas that integrate these basic functions mature later [42]. We could speculate that, without the necessary inhibitory processes to support decision-making, children and adolescents may be more susceptible to poor health-related choices, especially when evaluating nutritional cues in an obesogenic environment [43].

Diet type, physical activity, socio-demographic conditions, comorbidities and underlying brain mechanisms may mediate or moderate the relationship between neurocognitive functioning and obesity. Therefore, children and adolescents with attention deficit disorder require more help and support from adults in many daily life situations related to managing diet, activity, and the ability to solve emotional problems.

A small number of participants in our preliminary study required a uniform health status; therefore, only children with an isolated obesity diagnosis, without comorbidities and metabolic complications, were included. This approach allowed us to eliminate the possible influence of other diseases on the test outcomes. Our results clearly indicate a link between obesity and attention disorders; however, they do not allow for determining its cause-and-effect nature. Do attention deficits contribute to obesity, or does obesity itself contribute to attention deficits? The study by Pearce et al. shows that children with a normal body weight but a high familial risk of obesity have poorer executive functions even before they become obese, suggesting that some cognitive difficulties may precede, and not only result from, obesity [44]. It seems likely that attention disorders may facilitate excessive weight gain, but the increasing fat mass content may, in turn, exacerbate attention disorders due to disturbances in adipose tissue secretory functions. As an example, one of the best-known and functionally important adipokines is leptin. It appears that dysregulated leptin secretion, as observed in obesity, may contribute not only to metabolic complications but also to hippocampal synaptic disturbances and an increased risk of cognitive impairment [10]. Additionally, Sadler et al. indicate that adolescents with metabolic syndrome, or its components (e.g., insulin resistance, dyslipidemia), exhibit not only reduced attention, memory, and cognitive control, but also structural brain alterations. These alterations included gray matter volume, cortical thickness, white matter integrity, and modifications in functional neural connectivity, particularly in regions responsible for reward processing and cognitive control [45]. Neuropsychological impairments observed in youths with insulin resistance or type 2 diabetes are thought to result from a combination of factors, including chronic hyperglycemia, systemic inflammation, vascular dysfunction, and disrupted insulin signaling within the brain [46,47]. However, the majority of studies are cross-sectional in nature, making it impossible to distinguish cause from effect with certainty. Whether these metabolic disturbances cause cognitive impairments or vice versa is still unclear. It is also worth noting that both metabolic and cognitive conditions may be influenced by shared underlying variables, such as lifestyle, genetics, or environmental factors. Although our study included children with normal metabolic test results, excess adipose tissue itself causes changes in the adipokine profile, which were not examined in our study.

The results obtained indicate that it is reasonable to continue research in this field. The use of child-friendly tasks in research, including the introduction of computer tests such as MOXO CPT, may help to better understand attention processes in children with obesity. Although it seems that girls with obesity might be more affected by attention disturbances than boys, we would approach this conclusion with some caution. When using a computer-based method, such as CPT, the possibility that gender may influence test results should be taken into consideration. The question arises as to whether boys will perform relatively better than girls in computerized tests, even in the case of impaired attention functioning. In future studies, it would be worthwhile to significantly increase the size of the study group. This should not only allow us to explain the relationship between gender and the CPT results, but also to expand the analysis and assess the importance of other factors, such as the severity of obesity or the occurrence of complications, including dyslipidemia, dysglycemia, insulin resistance, or fatty liver disease, which was not possible in the current study. Additionally, evaluating selected adipokines would provide further information. Environmental influences, including detailed socioeconomic status and lifestyle-related variables such as sleep quality, physical activity, dietary habits, and home environment, should also be considered in future large-scale research.

Several limitations of this preliminary study need to be acknowledged. Relatively small sample size reduces statistical power and limits the generalizability of the findings. This issue is especially relevant for sex-stratified analyses, which should be interpreted with caution. Although participants were recruited consecutively from clinical settings, selection bias cannot be fully excluded. Additionally, detailed information on socioeconomic factors was not available; therefore, the homogeneity of both groups cannot be fully confirmed, which may have also affected the results obtained. Similarly, lifestyle-related variables were also not collected, and we cannot exclude their potential influence on the attention evaluation outcome. Regarding statistical analysis, no correction for multiple comparisons was applied, which increases the risk of a type I error, while borderline *p*-values should be interpreted in the context of limited statistical power. Although no extreme outliers were detected, some deviations from normality may have affected the performance of nonparametric tests. The cross-sectional design also precludes causal interpretations. Finally, due to the limited sample size, multivariable analyses and exploratory associations between metabolic and attention parameters could not be performed. As this study was preliminary in nature, our findings warrant replication in larger, more diverse cohorts and should be interpreted as a basis for future, more comprehensive investigations.

## 5. Conclusions

Attention efficiency appears to be reduced in children with obesity—particularly in girls—compared with their healthy peers. This may influence the way they analyze reality, make decisions, or initiate actions. Assessment of attention parameters in this group of patients could be valuable in the context of planning and implementing therapeutic interventions. We hypothesize that children with coexisting obesity and impaired attention functioning would probably require more assistance in following daily behavioral and nutritional recommendations.

The computerized continuous performance test may support clinical decision-making. However, interpreting its results may require caution depending on the child’s gender.

Overall, the present results should be viewed as preliminary and as a foundation for more comprehensive future large-scale studies, including detailed analysis of metabolic, environmental, and lifestyle-related variables.

## Figures and Tables

**Table 1:** Characteristics of children in the study and control groups.

Variable	Study Group Children with Obesity (*n* = 23)	Control Group Healthy Children (*n* = 48)
**Age [years]**		
Mean ± SD	11.27 ± 3.02	11.39 ± 2.67
Min./Max.	6.60/16.90	6.50/17.50
**BMI [kg/m^2^]**		
Mean ± SD	30.90 ± 6.05	17.29 ± 2.33
Min./Max.	22.38/44.58	14.0/22.80
**BMI z-score**		
Mean ± SD	2.42 ± 0.40	−0.29 ± 0.63
Min./Max.	2.01/3.24	−1.00/0.97

BMI, body mass index.

**Table 2:** Comparison of MOXO CPT results in children with obesity and healthy children.

Indicator of Attention	Children with Obesity (*n* = 23)		Healthy Children (*n* = 48)		Statistical Values	
	Mean Value	SD	Mean Value	SD	*p*	*r_c_*
Sustained attention	−0.14	1.34	0.57	0.77	0.074	-
Timing	−0.69	1.57	0.01	1.29	0.024	0.33
Hyperactivity	−0.91	3.28	1.02	1.00	0.001	0.48
Impulsivity	−1.20	1.34	−0.10	0.98	<0.001	0.50

U Mann–Whitney test; Glass biserial correlation coefficient.

**Table 3:** MOXO CPT results depending on gender within the group of children with obesity and the group of healthy children.

	Girls		Boys		Statistical Values	
	Mean Value	SD	Mean Value	SD		
**Children with obesity**	*n* = 12		*n* = 11		*p*	*r_c_*
Sustained attention	−0.13	1.22	−0.16	1.52	0.695	-
Timing	−0.88	1.66	−0.48	1.52	0.260	-
Hyperactivity	−1.38	3.86	−0.40	2.60	0.413	-
Impulsivity	−1.87	1.19	−0.48	1.13	0.013	0.60
**Healthy children**	*n* = 20		*n* = 28		*p*	*r_c_*
Sustained attention	0.65	0.68	0.51	0.84	0.268	-
Timing	0.13	1.66	−0.08	0.98	0.188	-
Hyperactivity	1.07	1.26	0.98	0.80	0.369	-
Impulsivity	−0.16	0.90	−0.06	1.04	0.530	-

U Mann–Whitney test; Glass biserial correlation coefficient.

**Table 4:** MOXO CPT results in girls and boys with obesity compared to healthy ones.

	Children with Obesity		Healthy Children		Statistical Values	
	Mean Value	SD	Mean Value	SD		
**Girls**	*n* = 12		*n* = 20		*p*	*r_c_*
Sustained attention	−0.13	1.22	0.65	0.68	0.035	0.45
Timing	−0.88	1.66	0.13	1.86	0.024	0.48
Hyperactivity	−0.49	2.47	1.07	1.26	0.012	0.53
Impulsivity	−1.87	1.19	−0.16	0.90	<0.001	0.75
**Boys**	*n* = 11		*n* = 28		*p*	*r_c_*
Sustained attention	−0.16	1.52	0.51	0.84	0.463	-
Timing	−0.48	1.52	−0.08	0.98	0.512	-
Hyperactivity	−0.40	2.60	0.98	0.80	0.105	-
Impulsivity	−0.48	1.13	−0.06	1.04	0.268	-

U Mann–Whitney test; Glass biserial correlation coefficient.

**Table 5:** MOXO CPT results by age groups among children with obesity and healthy children.

	Age Below 11 Years		Age Above 11 Years		Statistical Value
	Mean Value	SD	Mean Value	SD	
**Children with obesity**	*n* = 12		*n* = 11		*p*
Sustained attention	−0.22	1.56	−0.05	1.12	0.786
Timing	−0.44	1.88	−0.97	1.17	0.525
Hyperactivity	−1.36	4.01	−0.42	2.35	0.608
Impulsivity	−1.26	1.64	−1.14	0.98	0.928
**Healthy children**	*n* = 26		*n* = 22		*p*
Sustained attention	0.60	0.69	0.53	0.88	0.591
Timing	0.25	1.13	−0.28	1.43	0.420
Hyperactivity	1.08	0.94	0.94	1.09	0.901
Impulsivity	0.09	0.97	−0.33	0.96	0.131

U Mann–Whitney test.

## Data Availability

The data analyzed during this study are available upon reasonable request.

## Abbreviations

The following abbreviations are used in this manuscript:

CPT	Continuous performance test
BMI	Body mass index
ADHD	Attention deficit hyperactivity disorder
TSH	Thyrotropin
FT4	Free thyroxine
ALT	Alanine aminotransferase
AST	Aspartate aminotransferase
SD	Standard deviation
